# Oxidation of Flame Retardant Tetrabromobisphenol A by a Biocatalytic Nanofiber of Chloroperoxidase

**DOI:** 10.3390/ijerph16244917

**Published:** 2019-12-05

**Authors:** José Luis García-Zamora, Verónica Santacruz-Vázquez, Miguel Ángel Valera-Pérez, María Teresa Moreira, Diana L. Cardenas-Chavez, Mireya Tapia-Salazar, Eduardo Torres

**Affiliations:** 1 Centro de Química, Benemérita Universidad Autónoma de Puebla, Puebla 72570, Mexico; zamora422@hotmail.com; 2 Facultad de Ingeniería Química, Benemérita Universidad Autónoma de Puebla, Puebla 72570, Mexico; 3 Departamento de Investigaciones en Ciencias Agrícolas, Benemérita Universidad Autónoma de Puebla, Puebla 72570, Mexico; valeraperezmiguelangel@gmail.com; 4 Department of Chemical Engineering, CRETUS Institute, Universidade de Santiago de Compostela, Santiago de Compostela, E-15782 Galicia, Spain; maite.moreira@usc.es; 5 Tecnologico de Monterrey, School of Engineering and Science, Atlixcayotl 5718, Reserva Territorial Atrixcayotl, Puebla 72570, Mexico; diana.cardenas@tec.mx; 6 Facultad de Ciencias Biológicas, Universidad Autónoma de Nuevo León, Pedro de Alba, Ciudad Universitaria, San Nicolás de los Garza 66451, Mexico; mireya.tapiasl@uanl.edu.mx

**Keywords:** environmental biocatalysis, flame retardant degradation, micropollutants

## Abstract

Background: Tetrabromobisphenol (TBBPA), a flame retardant compound, is considered a ubiquitous pollutant, with potential impact on the environment and human health. Several technologies have been applied to accelerate its degradation and minimize environmental impacts. Due to its aromaticity character, peroxidase enzymes may be employed to carry out its transformation in mild conditions. Therefore, the purpose of this work was to determine the capacity of the enzyme chloroperoxidase (CPO) to oxidize TBBPA in several water samples. Methods: The oxidation capacity of CPO was evaluated in catalytic conditions using water samples from surface and groundwater, as well as effluents from wastewater treatment plants. The biocatalytic performance of CPO was improved due to its immobilization on nanofibers composed of polyvinyl alcohol and chitosan (PVA/chitosan). Results: Free and immobilized CPO were able to transform more than 80% in short reaction times (60 min); producing more biodegradable and less toxic products. Particularly, the immobilized enzyme was catalytically active in a wider range of pH than the free enzyme with the possibility of reusing it up to five times. Conclusions: The biocatalytic oxidation of TBBPA under environmental conditions is highly efficient, even in complex media such as treated effluents of wastewater treatment plants.

## 1. Introduction

Flame retardants (FRs) are compounds or mixtures of compounds that are incorporated primarily in plastics, textiles, wood, electronic circuits and other materials to prevent, reduce and retard the flammability or spread of flames across a surface when exposed to a low energy ignition source [1]. Consequently, FRs are widely distributed in all types of constructions: Public buildings, shopping and recreation centers, airports, schools, etc. They are also present in homes in products such as carpets, certain fabrics for upholstery and curtains, in coatings, construction elements and furniture of industrial origin, as well as in a multitude of household appliances [2]. According to their chemical nature, FRs are classified as phosphorous, nitrogen, inorganic or halogenated; the latter are the most widely used products worldwide, accounting for 45% of total production [2].

The global production of FRs in 2016 was estimated at 2.6 million metric tons with an annual growth of 5.4% over the last 30 reference years. This growth is related to the increased use of polymer-based materials and by the strict fire regulation. There are more than 75 different types of brominated retardants [3], of which, in terms of current or historical volume of production and use, the most relevant are polybrominated biphenyls (PBB), hexabromocyclododecane (HBCD), polybromodiphenyl ethers (PBDE) and tetrabromobisphenol A (TBBPA), this latter being the most commonly used. Due to its intensive use and its physicochemical properties, the ubiquitous presence of this pollutant not only in different environmental compartments, but also its potential of bioaccumulation in living organisms, has been documented. Several studies have found detectable amounts of TBBPA in the air (at production sites, indoor and outdoor) [4,5], soil and sediments (including activated sludge) [6,7,8], water (surface and groundwater, including landfill leachates and municipal wastewater effluent) [9,10], foodstuffs (processed and unprocessed) [11,12] and wildlife (aquatic and terrestrial) [13,14].

TBBPA is a persistent organic compound with a low rate of chemical and biological degradation [15,16]. In addition to being persistent, TBBPA is a mobile, bioaccumulative and toxic compound. Depending on the type of soil, temperature, humidity, and soil composition, approximately 17–90% of the TBBPA is degraded in the soil depending if aerobic or anaerobic metabolism is taking place [17]. Regarding toxicity, TBBPA has been reported as a potential immunotoxic, neurotoxic, endocrine disruptor [18,19]. According to several authors, wastewater treatment plants (WWTPs) are the main sources of TBBPA contamination since they receive all types of urban water, and especially because the WWTP treatment scheme has not been designed to eliminate these types of pollutants, but to reduce the biochemical oxygen demand (BOD) and nutrients [20,21,22].

Several technologies have been proposed for the removal of persistent organic pollutants (POPs) [23,24,25]. Advanced oxidation processes (AOP) and biological treatments are considered very promising green technologies for the degradation of hazardous compounds into non-toxic byproducts. Biocatalysis is a potential degradation technology that involves the POPs’ transformation into less toxic and more biodegradable products employing oxidative enzymes such as laccases and peroxidases [26,27,28]. Biocatalytic transformations are sustainable processes because they are conducted under mild reaction conditions, generally without toxic agents, they do not generate sludge as a side product and the energy demand of the process is low. While the low operational stability of enzymes is the main limitation of this technology, their immobilization in diverse organic, inorganic, and hybrid materials often improves their biocatalytic performance. Through immobilization, the thermostability, working temperature, and pH ranges might be extended [29,30]. In this sense, the nanoarrays of enzymes immobilized on the surface of nanomaterials constitute a group of state-of-the-art immobilized biocatalysts, with biocatalytic properties superior to those of conventionally immobilized derivatives [31,32]. Recent advances in nanomaterials open up the possibility of improving biocatalysis, since some nanostructured materials (mesoporous supports, nanoparticles, nanofibers, and nanotubes) have demonstrated their efficiency in the immobilization and performance improvement of enzymes [33,34]. These materials have high stability and a large surface area, which makes it possible to increase the enzyme load in the nanomaterial [35].

Chloroperoxidase (CPO) from *Caldaromyces fumago* is an oxidative enzyme of great interest in research due to its potential applications in multiple areas such as remediation. CPO is able to biotransform pollutants with different chemical nature, for example pharmaceuticals [26,36], organochlorides [37] and organophosphorus pesticides [38], azo dyes [39], and heterocyclic and polycyclic compounds [40]. This wide substrate variability makes it an excellent biocatalyst for transformation studies of organic micropollutants. In the present work, the biocatalytic transformation of TBBPA by CPO immobilized in nanofibers is reported. In order to identify the potential of this enzyme, a comparative study with the free enzyme was proposed in order to determine the technological feasibility of both alternatives, in particular the kinetic parameters, the reaction products, the biodegradability index, and the toxicity of the oxidation products. The catalytic capacity of immobilized enzymes was evaluated in several environmental water matrices, such as surface water, groundwater, and effluent samples from two WWTPs from Puebla city, Mexico, to test the capacity of the enzyme to operate in complex media.

## 2. Materials and Methods

### 2.1. Chemicals

Tetrabromobisphenol-A (TBBPA), chitosan, polyvinyl alcohol (PVA), and glutaraldehyde were provided by Sigma-Aldrich (St. Louis, Missouri, USA). Chloroperoxidase from *Caldariomyces fumago* (CPO) was purchased from Alltaenzymes (Edmonton, AB, Canada) and presented the following features: An R_Z_ of 1.4 (the R_Z_ value is the absorbance ratio A_403_/A_275_, which indicates the purity level of the CPO solution; a value of 1.4 is considered a high-purity enzyme), and a maximum halogenating specific activity of 22,000 min^−1^ for monochlorodimedone. Buffer salts, isopropyl alcohol (HPLC grade), methylene chloride (HPLC grade), sodium sulfate, hydrogen peroxide, and sodium chloride were purchased from J.T. Baker (Phillipsburg, NJ, USA).

### 2.2. Enzymatic Activity in Buffer Systems

CPO catalytic activity towards TBBPA was determined in a 1 mL reaction volume containing 260 nM of the enzyme and 10 µM of the TBBPA in 60 mM phosphate buffer (pH 3, 25 °C). All the reactions were initiated by adding hydrogen peroxide 0.1 mM. The control sample contained the aforementioned components except the enzyme. The reaction progress was monitored for 10 min in function of the change in the substrate peaks by HPLC and UV-vis detection. Remaining substrate concentration was measured every minute, after stopping the reaction with 2-propanol (1 mL). A standard curve for TBBPA was previously prepared to transform the peak areas. The conversion was calculated as follows:

(1) $$\%\;Conversion=\frac{\left(C_{0}-C_{t}\right)}{C_{0}}*100\%$$

where *C*_0_ and *C_t_* are the initial and remaining TBBPA concentration at different reaction times.

To determine the reaction constant, the decrease in TBBPA concentration was plotted against time, and the data were fitted to a first-order reaction using Origin 9.0 software (Originlab Corporation, Northampton, MA, USA). The reported values correspond to the average value of three replicates.

### 2.3. Preparation and Characterization of Nanofibers

Chitosan/polyvinyl alcohol nanofibers were electrohilated from a 3% *w*/*v* chitosan solution in acetic acid (1 M), and a 10% *w*/*v* solution of polyvinyl alcohol in a 1:1 ratio, using needle caliber 22 G (0.7 mm × 32 mm). The electro-spinning parameters were 28 kV flow rate 0.2 mL h^−1^ and 100 rpm collection, and three parameters were varied, the collection distance (15 to 25 cm), the collection time (30 to 60 min), and electric potential (20 to 30 kV). The nanofibers were then collected for 1 h on an aluminum foil, before being dried for 48 h in a desiccator.

#### 2.3.1. Scanning Electron Microscopy

The electrospun nanofibers (1 cm^2^) were subjected to a gold bath under high vacuum for 1 min using a unit of devastated and bombardment of Denton Vacuum Desk V brand model. The micrographs were obtained at 500×, 1000×, 3000×, 5000×, and 10 000× with an acceleration voltage of 25 kV in a JEOL scanning electron microscope model JSM-6610 equipped with Smile ViewTM software. The nanofiber diameter was determined using ImageJ v1.51j8 image processing software.

#### 2.3.2. Fourier Transform Infrared Attenuated Total Reflectance

The infrared spectra of the electro-spun nanofibers were obtained with a FTIR/ATR Perkin Elmer Spectrum 100 model equipped with a germanium crystal. The infrared spectra were collected with the SpectrumTM 10 software included in the equipment, from 600 to 4000 cm^−1^.

#### 2.3.3. Differential Scanning Calorimetry

The thermal analysis of the nanofibers was carried out in a DSC TA Instruments model 2010 equipped with a flow meter and calibrated with indium (T_m_ = 429.75 K). In one of the two aluminum cells, 2 mg of the fibers were added, while the other cell was empty. The analysis was performed under a nitrogen atmosphere with a flow of 70 mL min^−1^, with a heating ramp of 10 °C min^−1^ in the temperature range from 25 to 600 °C. The glass transition (T_g_) and melting (T_m_) temperatures, as well as the enthalpies of fusion, were calculated using the Universal Analysis 2000 v4.5A software.

### 2.4. Enzyme Immobilization

Prior to CPO immobilization, the nanofibers were cross-linked using glutaraldehyde vapors for 48 h at room temperature. Nanofiber was then washed 10 times with distilled water to remove the glutaraldehyde excess. Subsequently, 1 cm^2^ of activated fiber was placed in contact with 2.3 µM CPO in 1 mL of 60 mM phosphate buffer (pH 3.0) for 24 h at 4 °C under gentle agitation. After the incubation time, the nanofibers were washed 10 times with buffer until no enzymatic activity was detected in the washing solution (halogenation of thionin acetate [41]). The amount of CPO immobilized was estimated as the difference of the catalytic activity before and after CPO adsorption (recovered enzyme in the washing solution). The final enzyme preparation was kept in 1 mL of 60 mM phosphate buffer at pH 3. The CPO–nanofiber catalytic activity was determined as described above. The removal of TBBPA was evaluated by both conversion and adsorption incubating the nanofibers. For the former, the nanofibers were incubated in the reaction system in the absence of hydrogen peroxide.

The conversion mediated by the free enzyme and CPO–nanofibers was determined in the pH range of 3–7. Meanwhile, immobilized enzyme recyclability was tested at pH 3. For this purpose, the reaction was carried out for 30 min, after which the nanofibers were separated from the reaction mixture and incorporated into a new reaction mixture for reuse. The TBBPA conversion was determined by HPLC as previously described.

### 2.5. HPLC Analysis

TBBPA concentration was measured employing a Perkin Elmer HPLC system (Hopkinton, MA, USA) equipped with a UV-vis detector, binary pump system, an injection loop of 20 µL and a reversed-phase C-18 column (Phenomenex Luna^®^, Torrance, California, USA, 5 µm). Changes in peak areas occurred at 220 nm using a pH 2 acetonitrile–phosphate buffer solvent mixture at a flow rate of 0.7 mL/min.

### 2.6. Product Extraction

For identification of the oxidation products by mass spectrometry, the reaction was carried out on a 100 mL scale. The products were extracted using a 200 mg Lichrolut^®^ RP-18 SPE cartridge (particle size of 40–63 µm), previously conditioned with 10 mL of methanol followed by 6 mL of water and a flow of 2 mL min^−1^. For the washing step, 10 mL of water followed by 10 mL of a 20/80 *v*/*v* methanol-water solution were applied. The cartridges were then vacuum dried for 5 min. The reaction products were eluted by gravity with 5 mL of methanol, dried in a gentle stream of nitrogen and reconstituted in 1 mL of methanol for identification.

### 2.7. Products Identification

Reaction products were identified by LC-MS (Chromatograph Series 1260) coupled to ESI-Q-TOF-MS (6520, Agilent Technologies, Santa Clara, California, USA). The separation was performed on a ZORBAX SB C18-Selec column (4.1 × 100 mm, 5 μm). The parameters of the ESI source were the following: Negative ionization mode, fragmenter voltage: 175 V, capillary voltage: 3500 V, gas temperature: 350 °C, N_2_ flow: 11 L min^−1^, nebulizer pressure: 60 psi, and the flow rate: 0.7 mL min^−1^.

### 2.8. Enzymatic Oxidation of TBBPA in Environmental Water Samples

The CPO oxidative capability was determined in several water samples (wastewater effluent produced from two WWTPs in Puebla City, Mexico, surface water from Nexapa and Chapa Chapa rivers, a lagoon, and groundwater). The samples were first filtered to remove suspended solids and particulate matter and stored at 4 °C prior to use. The samples were analyzed by standard methods for physicochemical parameters (pH, conductivity, temperature, chemical oxygen demand (COD), BOD, concentrations of Ca^2+^, Fe^2+^, Ni^2+^, Mg^2+^, SO_4_^2−^, NO_3_^1−^, PO_4_^3−^, and free chlorine. Water analysis is summarized in the Supplementary Material (Table S1). The water samples were then spiked with 10 µM of TBBPA, and the transformation was assayed as described for the model systems. Three replicate experiments were performed for all samples.

### 2.9. Biodegradability Determination

The BOD_28_/OD ratio was selected as the biodegradability index for this study. The BOD values after 28 days were determined employing BOD bottles with glass stoppers, and standard Winkler titration [42]. The inoculum was taken from a sample of surface water taken in the Nexapa River in the state of Puebla, Mexico.

### 2.10. Toxicity Assays

For the toxicity tests, *Artemia salina* was chosen as the model organism due to the ease and speed of the assays. In addition, it is an important model for marine ecosystems, where TBBPA has been detected. Artificial marine water was prepared by dissolving the salt mixture Instant Ocean^®^ in distilled water (29.9 g L^−1^). It was used as negative control and diluent for the testing solutions. *Artemia salina* cysts (Biogrow, Proaqua^®^) were first hatched to obtain the nauplii. The cysts were decapsulated with a hypochlorite solution [43], washed in tap water and placed into a glass container with marine water. They were kept under illumination and constant aeration for 24 h at 28 °C. Toxicity tests were performed in 96-well plates [44], where each well was filled with 20 μL of marine water containing 10 nauplii and 230 μL of TBBPA stock solution or media reaction containing its degradation products after enzymatic treatment. The final contaminant concentrations per well were 1, 2.5, 5, 10, 15, 20, 25, 30, 35, or 40 µg L^−1^. This concentration range was established based on the basis of preliminary experiments in our laboratory (data not shown). All tests were carried out in triplicate at two temperatures: Room temperature (20.5 to 23.5 °C) and 28 °C. Survival was recorded after 24 and 48 h of exposure. Positive control containing potassium dichromate was also included in the experiments. Mean lethal concentration (LC_50_) was determined by nonlinear regression for each test and then compared to calculate an average. These regressions were obtained using Origin 9.0 software.

## 3. Results

### 3.1. Nanofiber Preparation

#### 3.1.1. Morphology

**Figure 1:**
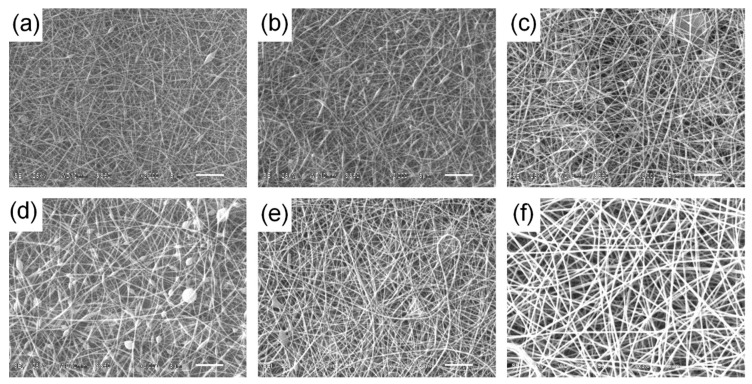
Morphology of produced nanofibers at different collection distance, collection time, and voltage (**a**) 20 cm, 60 min, 30 kV; (**b**) 15 cm, 30 min, 25 kV; (**c**) 20 cm, 30 min, 25 kV; (**d**) 20 cm, 60 min, 20 kV; (**e**) 20 cm, 60 min, 25 kV; (**f**) 15 cm, 60 min, 20 kV.

The results of nanofiber preparation under different time and distance of collection, as well as different voltage conditions are shown in Table 1 and Figure 1. On the one hand, the presence of bulbs (beads) can be observed, which are common defects in the preparation process attributed to a number of effects relative to surface tension, which turns Taylor’s cone into droplets; to the repulsion of electrostatic charges on the surface of the polymer jet; and to low electrical potentials, promoting the accumulation of material at the outlet of the injector by not counteracting the surface tension of the polymer [45]. That is why the increase of the electrical potential favors the repulsion of electrostatic charges along the Taylor cone and the surface of the jet helping the formation of smaller diameter homogeneous fibers. It can be concluded that the best conditions for the final homogeneity of the nanofibers correspond to a collection time of 60 min, collection distance of 15 cm and 20 kV electric potential (sample F, Table 1, Figure 1), so that there is no accumulation of material along the surface.

#### 3.1.2. FTIR Characterization

**Figure 2:**
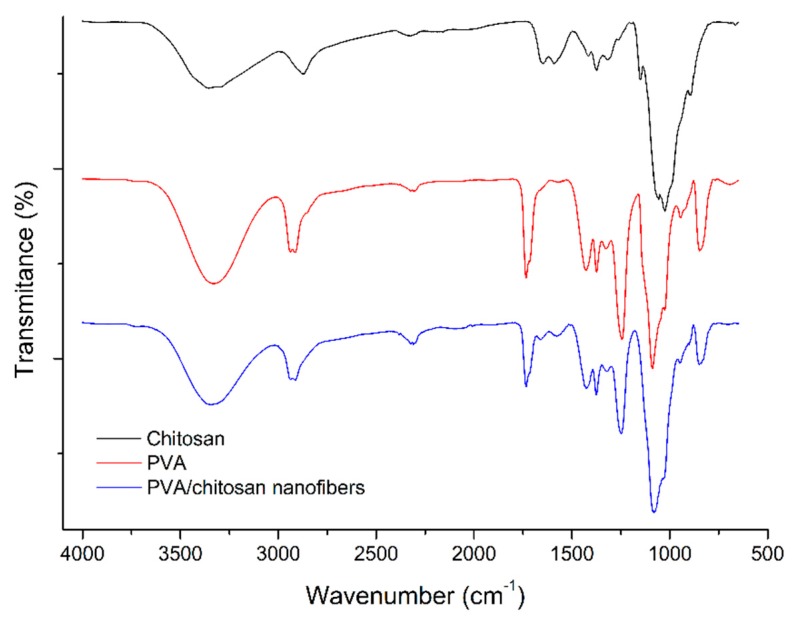
FTIR spectra for polyvinyl alcohol (PVA)/chitosan nanofibers and their comparison to the polymer precursors.

PVA/Chitosan nanofibers show combined spectroscopic characteristics of their components (Figure 2, Table S2). On the one hand, the stretching bands for CO at 1747 cm^−1^ and COOC at 1248 cm^−1^ are clearly present in the nanofiber, since PVA is the main component [46]{Choo, 2016 #47}. In addition, some of the functional groups of the chitosan can be distinguished at 1644 cm^−1^ and 1582 cm^−1^ assigned to bending and stretching vibrations of NH_2_ and NH, respectively [47,48]. In addition, the vibrations of the CH_3_CO group at 893 cm^−1^ were observed [49]. Furthermore, the decrease in the intensity of the band corresponding to the stretching of the CH_2_ group (2915 cm^−1^) can be seen in comparison with the spectrum of the pure PVA spectrum, attributed to the presence of hydrogen bonds between the polymers [47].

#### 3.1.3. Calorimetry Characterization

**Figure 3:**
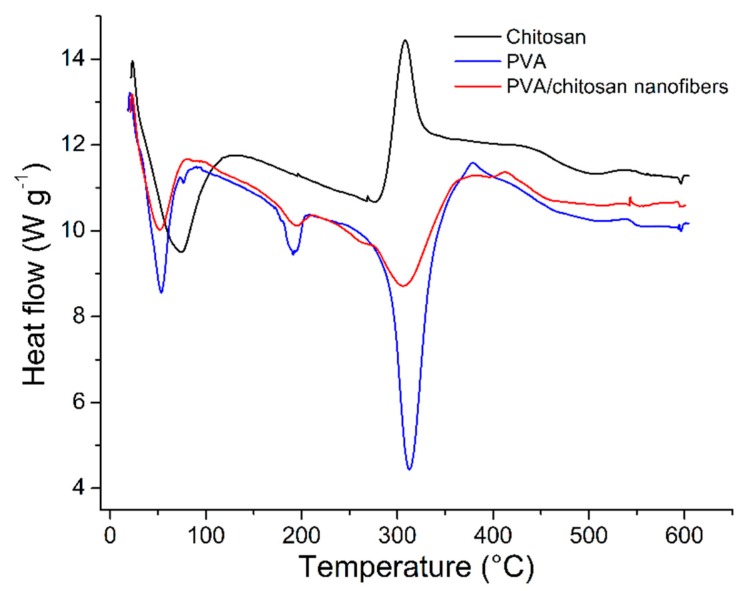
Thermograms of PVA/chitosan nanofibers and their polymer precursor.

The thermogram of the PVA/chitosan nanofiber shows changes compared to the PVA thermogram, which is attributed to the presence of chitosan as a second component (Figure 3). The T_g_ value of the mixture showed a minor change with respect to the neat PVA (from 53.7 to 51.9 °C); in addition, changes in fusion (from 190 to 193 °C) and degradation (from 313 to 309 °C) temperatures were observed; this last transition showed a decrease in the associated heat of degradation, from 648 to 227.3 J/g, attributed to the exothermic heat associated with the degradation of the chitosan fraction present in the nanofiber, which occurs in the same temperature range (311 °C).

### 3.2. Enzyme Immobilization

**Figure 4:**
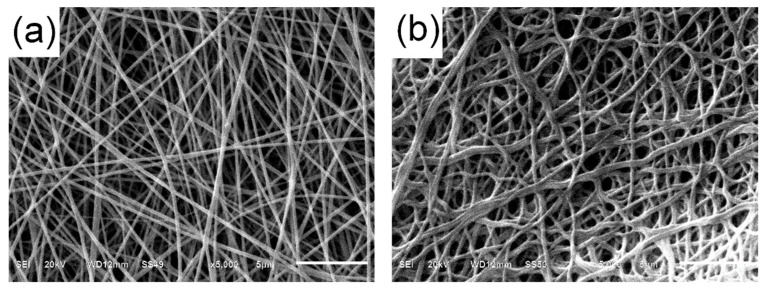
Morphology of a glutaraldehyde-activated nanofiber before (**a**) and after (**b**) chloroperoxidase immobilization.

Prior to incubation with the enzyme, the nanofibers were exposed to glutaraldehyde vapors to activate the surface, with the aim of modifying the amino groups of chitosan to incorporate a reactive group of aldehyde. Figure 4 shows the morphology of the nanofibers after incubation with glutaraldehyde, where it can be observed that the nanofibers remain without apparent change. However, nanofibers change their appearance noticeably when the enzyme is immobilized, showing a larger diameter and some degree of cross-linking (Figure 4). The amount immobilized was 14.8%, producing a nanofiber with an enzyme load of 340.4 nmol/cm^2^. Digital image analysis revealed a decrease in surface area after immobilization from 96.6 m^2^/g to 68.7 m^2^/g, representing a reduction of approximately 28.8% of the surface area.

### 3.3. Oxidation of TBBPA by the Free and the Immobilized CPO

**Figure 5:**
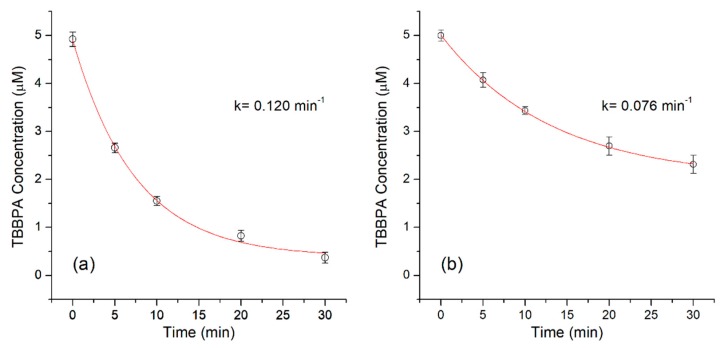
Time course oxidation of tetrabromobisphenol (TBBPA) by (**a**) free and (**b**) nanofiber immobilized chloroperoxidase (CPO).

TBBPA has been recognized as a persistent organic compound, with low degradability and high environmental impact. Figure 5 shows that the CPO is capable of oxidizing 68.49% ± 1.92% of the initial TBBPA concentration in 10 min. The conversion reaches up to 93.5% ± 2.23% after 30 min of reaction. The data were adjusted to a first order model, which resulted in a kinetic constant (k) of 0.12 ± 0.0093 min^−1^ (R^2^ 0.98). For the immobilized enzyme, the conversion was 54.93% ± 3.98 % in the first 30 min of reaction; this removal value was increased to 89.27% ± 4.91 % after 90 min of reaction, with a k-value of 0.076 ± 0.005 min^−1^ (R^2^ 0.99), lower than that of the free enzyme. Similar conversion results were reported by Xu et al. [50], with the immobilization of horseradish peroxidase (HRP) on nitrocellulose nanofibers, achieving a TBBPA conversion of 95.9% in 3 h.

As for the oxidation of TBBPA by free enzymes, HRP and laccase reached around 100% removal for TBBPA in 30 min [51,52]. It is well known that immobilization reduces the catalytic activity of enzymes by different factors from geometric up to diffusional limitations [29]. Both the conversion and the time to reach it are within the range reported for other chemical or biological elimination methods. The advantage of a chemical treatment is that it can lead to the mineralization of the compounds, although it requires more energy or reaction time (up to 3 h according to Bao et al., 2015 [53]). The enzymatic oxidation can in a short time transform a recalcitrant and toxic pollutant, giving rise in many cases to products of higher biodegradability and lower toxicity.

#### 3.3.1. Product identification

The reaction products of TBBPA enzymatic oxidation by peroxidases and laccase have been identified, as well as those produced by chemical oxidation such as ozonation [52,54,55]. In this study, at least two reaction products could be identified (Table 2). The first with chemical formula C_9_H_10_Br_2_O_2_ and molecular weight of 309.9053 may correspond to three different compounds (Table 2). The second product corresponds to a product with chemical formula C_17_H_17_Br_3_O_2_ and with a molecular weight 493.8289. Both products showed the de-halogenation of TBBPA with subsequent reactions that may lead to the opening of one of the phenolic rings. The enzymatic oxidation of TBBPA produced other reaction products such as those reported using horseradish peroxidase, laccase, mostly with lower molecular weights, which indicates that a hydrolytic reaction took place after the enzymatic oxidation [51,52].

#### 3.3.2. Product Biodegradability and Toxicity

According to the OECD 301D specifications, the ratio of biochemical oxygen demand/oxygen demand (BOD_28_/OD) may represent the biodegradability index of a substance. Here, the experimental COD was used instead of the theoretical oxygen demand due to difficulty in separating and identifying all reaction products. A sample of non-adapted microorganisms from the River Nexapa was further employed as inoculum. The biodegradability index of the mixture of oxidized products was 10 times higher than that of TBBPA (Table 3). This result may be partially explained since TBBPA presents significant toxicity to the inoculum, inhibiting 80% of microbial respiration (Table 3). This fact also suggests that the enzymatic products are less toxic (26.5% of inhibition of microbial respiration). However, this assay is not considered as a real probe of toxicity; thus, another toxicity test must be performed to confirm it. For the study of the toxicity of the reaction products, the model organism *Artemia salina* has been widely used for its high sensitivity to chemical compounds, as well as for its easy availability, handling, and maintenance under laboratory conditions. *Artemia salina* shows highly reproducible and comparable responses to standardized test organisms such as *Daphnia magna*, *Vibrio fischeri*, and *Danio rerio*, among others [57,58].

**Figure 6:**
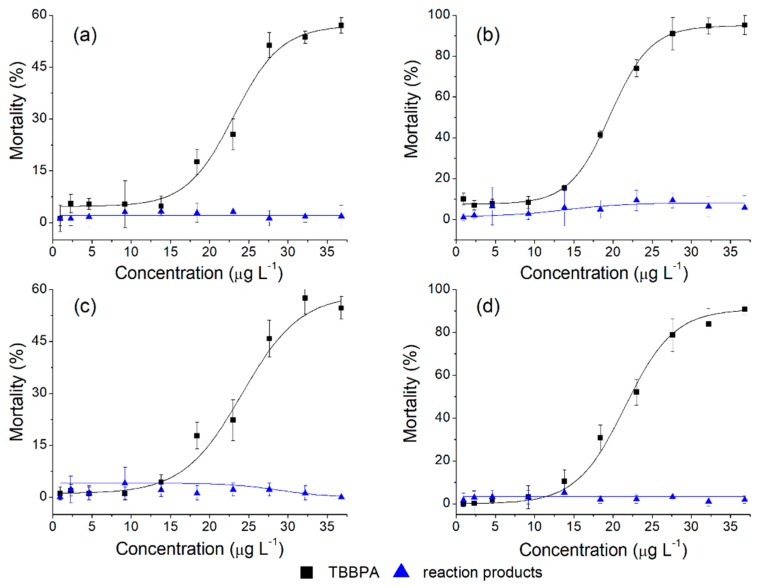
Dose-response curves for TBBPA in *Artemia salina* at 21 °C for 24 h (**a**) and 48 h (**b**); and 28 °C for 24 h (**c**) and 28 h (**d**).

The dose-response curves for exposure to temperature of 21 °C and 28 °C are presented in Figure 6. The LC_50_ of the TBBPA at 21 °C was 29.86 µg L^−1^ and 19.18 µg L^−1^ during 24 and 48 h of exposure, respectively. The maximum mortality of *Artemia saline* exposed for 24 h, at the highest concentration of TBBPA (36.8 µg L^−1^), was 57.7%, which increased to 98.2% at 48 h of exposure (Figure 6a,b). At a higher temperature (28 °C), the LC_50_ increased slightly to 31.91 µg L^−1^ and 24.47 µg L^−1^ for 24 and 48 h of exposure, respectively (*p* < 0.05).

In the case of the maximum concentration tested (36.8 µg L^−1^), the mortality increased from 54.7% to 90.8% corresponding to exposure periods of 24 and 48 h (*p* < 0.05) (Figure 6c,d). According to Nunes et al. (2006) [43], *Artemia* can survive at temperatures between 15 and 32 °C, and is best adapted to 26 °C. As can be seen in Figure 6, the toxicity of the enzymatic reaction products does not have a significant effect on the mortality of *Artemia saline* under identical test conditions: Temperatures, concentrations, and exposure times (*p* > 0.05). It can be therefore concluded that biocatalytic reaction products are significantly less toxic than their unmodified counterpart (*p* < 0.05).

#### 3.3.3. pH Profile and Recyclability Assays

**Figure 7:**
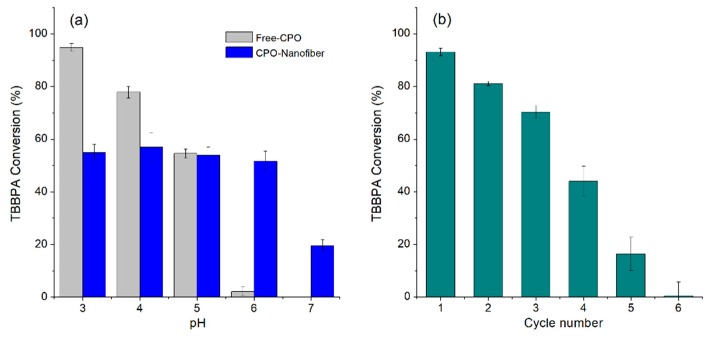
(**a**) pH profile of free and immobilized chloroperoxidase, and (**b**) recyclability of CPO immobilized on PVA/chitosan nanofibers.

One of the reasons to immobilize enzymes is to overcome the limitations shown in a soluble form. For free CPO, two of the determining factors in its eventual industrial application are its narrow pH profile (3–5) and the difficulty of reusing the enzyme in different treatment cycles. Figure 7a shows that the immobilization of the CPO in nanofiber widens the pH range at which it is active, while the soluble enzyme was active at 2–5, with an almost total loss of activity at pH 6 (2% remaining activity). The immobilized CPO did not show a pronounced change in its activity for a pH range between 3 and 6, still maintaining 20% of the activity at pH 7. Some reports of CPO immobilization have shown that the enzyme is sensitive to a pH above 5, since it suffers from irreversible loss of activity [39,59].

The fact that the incorporation in the nanofibers allows the activity at pH to be superior to 6 increases the potential of application of the enzyme in the different applications, which is a very positive aspect for its application in environmental biocatalysis since wastewater effluents normally have a neutral pH. It has been reported that immobilization with chitosan, silica derivatives, and other materials can modify the pH dependence of peroxidases by providing a favorable microenvironment for their catalytic activity [60,61,62].

Improved activity and stabilization or activity of enzymes by immobilization is often the result of a combination of physicochemical factors, such as enzyme inhibition, rigidification or distortion, pH gradients, substrate or product gradients, and partitioning of the substrates or products, which may greatly improve enzyme performance. In terms of recyclability, the enzyme was reused for up to five reaction cycles, though a significant loss of activity of approximately 50% was observed in the fourth cycle (Figure 7b). In the fifth cycle, the conversion was decreased to about 20%, which can be attributed to the loss of activity by inactivation by hydrogen peroxide as the main cause of the decrease in peroxidase activity reported in the literature [63,64].

### 3.4. Enzymatic Oxidation of TBBPA in Environmental Water Samples

Finally, the ability of the immobilized CPO to oxidize TBBPA was determined in several samples of drinking water, surface water (two rivers), a lagoon, groundwater, and treated wastewater produced from two treatment plants in Puebla, Mexico (Table S1 for physicochemical characterization). These water samples did not contain measurable quantities of TBBPA, except for one of the WWTP effluents, though at very low concentrations under the detection limit of the HPLC equipment. Therefore, TBBPA was spiked to all water samples at 10 µM. In addition, hydrogen peroxide and sodium chloride were added to a final concentration of 1 and 20 mM, respectively. It was also required to adjust the samples to pH 6. The results show that both free and immobilized enzymes maintain the ability to oxidize TBBPA in varied water matrices including treated effluents from the two treatment plants, despite having more drastic environmental conditions for enzymes such as high COD, salts, etc. The average conversion at 30 min was 50%, while at 90 min an average conversion of 90% was achieved (Table 4).

In conclusion, the nanobiocatalyst showed a high oxidative capacity towards TBBAP in simple and complex aqueous matrices such as treated wastewater, suggesting that this method could be considered as potential polishing technology to oxidize toxic and recalcitrant compounds. The results also indicated that the product biodegradability was higher and the toxicity lower, so the treatment also facilitates the biological attenuation of the effluent once discharged into natural water bodies.

## Figures and Tables

**Table 1:** Physicochemical parameters of the nanofibers produced.

Sample	Collection Distance (cm)	Collection Time (min)	Voltage (kV)	Average Fiber Diameter ^1^ (nm)
A	20	60	30	222.0 ± 21.9
B	15	30	25	184.4 ± 38.1
C	20	30	25	239.6 ± 56.3
D	20	60	20	224.0 ± 69.2
E	20	60	25	318.0 ± 48.7
F	15	60	20	212.9 ± 22.7

^1^ Digital image analysis.

**Table 2:** Reaction products of CPO-mediated oxidation of tetrabromobisphenol A.

Formula	*m*/*z*	Chemical Structure	Error (ppm)	Reference
C_15_H_12_Br_4_O_2_	543.7333	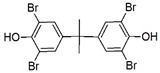 3,3’,5,5’-tetrabromobisfenol A	2.51	This work
C_9_H_10_Br_2_O_2_	309.9053	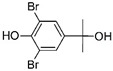 2-6-dibromo-4-(1-hydroxy-1-methylethyl) phenol	2.49	[52,53,55]
		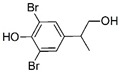 4-(2-hydroxyisopropyl)-2,6-dibromophenol		
		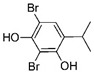 2,6-dibromo-4-isopropyl-3-hydroxyphenol		
C_17_H_17_Br_3_O_2_	493.8289	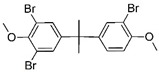 3,3’,5-tribromobisphenol A dimethyl ether	16.25	[54,56]

**Table 3:** TBBPA biodegradability test (before and after chloroperoxidase transformation).

Sample	BOD (mg O_2_/L)	COD (mg O_2_/L)	Biodegradability Index (%)	Inhibition ^1^ (%)
TBBPA	21.7	308.0	7.0	80.6
Reaction products	95.0	121.5	78.2	26.5

^1^ Inhibition was calculated as [100 − BOD_dextrose/glutamic acid_/BOD_dextrose/glutamic acid/TBBPA_].

**Table 4:** Oxidative capacity of PVA/Chitosan nanofibers in environmental water samples.

Water Source	TBBPA Conversion (%)	
	30 min	60 min
Buffer	54.93	89.27
Distilled water (no pH control)	52.87	92.46
Treated wastewater A ^1^	53.50	87.11
Treated wastewater B ^2^	58.62	95.23
Lagoon	53.87	91.39
Nexapa River	45.68	79.35
Chapa-Chapa River	57.31	86.75
Groundwater	54.86	90.07

^1^ From municipal WWTP from Puebla state, Mexico. ^2^ From municipal WWTP from Puebla city, Mexico.

## Supplementary Materials

The following are available online at https://www.mdpi.com/1660-4601/16/24/4917/s1, Table S1. Physicochemical characterization of environmental water samples; Table S2. FTIR characterization of nanofibers and their precursors.
