# Supplementary material for: Oxidation of Flame Retardant Tetrabromobisphenol A by a Biocatalytic Nanofiber of Chloroperoxidase
Source: Int J Environ Res Public Health. 2019 Dec 5;16(24):4917. doi: 10.3390/ijerph16244917 (PMC6950518; doi:10.3390/ijerph16244917)
Supplement: Supplementary file 1 [file ijerph-16-04917-s001.pdf]

Oxidation of flame retardant tetrabromobisphenol A by a biocatalytic nanofiber of chloroperoxidase

José Luis García-Zamora[1], Verónica Santacruz-Vázquez[2], Miguel Ángel Valera-Pérez[3], María Teresa Moreira[4], Diana L. Cardenas-Chavez[5], Mireya Tapia-Salazar[6] and Eduardo Torres[1]*

Table S1. Partial physicochemical characterization of environmental water samples.

Parameter	Groundwater	Surface water[1]			Treated wastewater[2]	
		Lagoon	River A	River B	WWTP A	WWPT B
pH	6.55	8.84	7.42	7.54	7.52	7.27
BOD (mg O_2/L)	2.0	3.0	20.0	5.5	160.0	37.0
COD (mg O_2/L)	5.0	8.5	832.0	16.5	781.5	104.0
TDS (m/L)	652.0	452.0	681.0	636.0	1120.0	1349.0
Conductivity (µS/cm)	921.0	593.0	1313.0	872.0	2260.0	1423.0
Free Chloride (mg/L)	0.09	0.05	0.0	0.165	0.14	0.05
NO_3^{1-} (mg/L)	4.05	27.45	0.0	39.70	80.0	268.00
PO_4^{3-} (mg/L)	0.68	1.78	3.5	0.51	45.0	106.50
SO_4^{2-} (mg/L)	96.0	74.0	175.0	137.0	95.0	97.0
Mg^{2+} (mg/L)	10.0	15.0	35.0	20.0	65.0	15.0
Ca^{2+} (mg/L)	0.0	103.5	160.0	10.0	200.0	0.0
Fe^{3+} (µg/L)	66.0	18.0	0.375	36.0	1310.0	--

[1] River A: Nexapa River and River B: Chapa-Chapa River; [2]WWTP A: from Puebla State and WWTP B: from Puebla city.

Table S2. FTIR characterization of nanofibers and their precursors.

Characteristic group	Wavenumber (cm^{-1})		
	Chitosan	PVA	Nanofibers
ν(NH_2) assoc. in primary amines and ν(OH) asooc. in pyranose ring	3362	–	3348
ν(OH) from intermolecular and intramolecular hydrogens bonds	–	3335	3348
ν(CH_2) in alkyl groups	–	2938	2938
ν(C-H) in pyranose ring and alkyl groups	2875	2915	2916
ν(C = O) as polyvinyl acetate residual	–	1734	1747
ν(C = O) as polyvinyl acetate residual	–	1722	1722
ν(NH_2) in $NHCOCH_3$ group (Amide I band)	1652	–	1665
ν(NH) in $NHCOCH_3$ group (Amide II band)	1593	–	1582
δ(CH_2) inCH2OH group	1418	1429	1429
$δ_s$ (CH_3) in $NHCOCH_3$ and methyl groups	1374	1374	1377
δ(C-H) in carbon chain	1316	1326	1324
ν (CO-O-C) as polyvinyl acetate residual	–	1245	1248
$ν_s$ (C-O-C) glycosidic linkage	1151	–	1147
ν(C-O) in C-OH groups	–	1088	1083
$ν_{as}$ (C-O-C) glycosidic linkage	1057	–	–
ν(C-O) in secondary OH group	1026	1026	1035
CH_3CO group in pyranose ring	895	–	905
ν(C-C) in carbon chain	–	848	852
